# PKCδ Sensitizes Neuroblastoma Cells to L-Buthionine-Sulfoximine and Etoposide Inducing Reactive Oxygen Species Overproduction and DNA Damage

**DOI:** 10.1371/journal.pone.0014661

**Published:** 2011-02-07

**Authors:** Barbara Marengo, Chiara De Ciucis, Roberta Ricciarelli, Mario Passalacqua, Mariapaola Nitti, Jean-Marc Zingg, Umberto M. Marinari, Maria A. Pronzato, Cinzia Domenicotti

**Affiliations:** 1 Giannina Gaslini Institute, Genoa, Italy; 2 General Pathology Section, Department of Experimental Medicine, University of Genoa, Genoa, Italy; 3 Biochemistry Section, Department of Experimental Medicine, University of Genoa, Genoa, Italy; 4 Centre of Excellence for Biomedical Research, University of Genoa, Genoa, Italy; City of Hope National Medical Center, United States of America

## Abstract

Neuroblastoma is a type of pediatric cancer. The sensitivity of neuroblastoma (NB) cancer cells to chemotherapy and radiation is inhibited by the presence of antioxidants, such as glutathione (GSH), which is crucial in counteracting the endogenous production of reactive oxygen species (ROS). We have previously demonstrated that cells depleted of GSH undergo apoptosis *via* oxidative stress and Protein kinase C (PKC) δ activation. In the present study, we transfected PKCδ in NB cells resistant to oxidative death induced by L-buthionine-S,R-sulfoximine (BSO), a GSH-depleting agent. Cell responses, in terms of ROS production, apoptosis and DNA damage were evaluated. Moreover, PKCδ activation was monitored by analyzing the phosphorylation status of threonine 505 residue, carrying out PKC activity assay and investigating the subcellular localization of the kinase. The cell responses obtained in BSO-resistant cells were also compared with those obtained in BSO-sensitive cells subjected to the same experimental protocol. Our results demonstrate, for the first time, that PKCδ induces DNA oxidation and ROS overproduction leading to apoptosis of BSO-resistant NB cells and potentiates the cytotoxic effects induced by BSO in sensitive cells. Moreover, PKCδ overexpression enhances the sensitivity of NB cells to etoposide, a well-characterised drug, commonly used in neuroblastoma therapy. Altogether our data provide evidence of a pro-oxidant role of PKCδ that might be exploited to design new therapeutic strategies aimed at selective killing of cancer cells and overcoming drug resistance. However, it becomes evident that a more detailed understanding of ROS-mediated signaling in cancer cells is necessary for the development of redox-modulated therapeutic approaches.

## Introduction

Neuroblastoma relapse is caused by a minimal residual disease, characterized by the presence of a small number of cancer cells in the blood and/or in the bone marrow that are resistant to conventional therapies. In this context, drugs capable of inducing apoptosis and the study of apoptotic mechanisms have generated a particular interest [1].

During programmed cell death, kinases such as p38 mitogen-activated protein kinase, c-Jun N-terminal kinase (JNK), extracellular signal-regulated protein kinase and protein kinase C (PKC) are regulated in a cell type-dependent manner [2].

PKC is a family of phospholipid-dependent serine/threonine kinases that regulate a wide variety of cellular functions [3]. The PKC family consists of at least eleven members that have been divided into three groups: conventional or cPKCs (α, βI, βII and γ) requiring calcium and diacylglycerol (DAG) for their activation, novel or nPKCs (δ, ε, η and θ) dependent on DAG but not on calcium and finally, atypical or aPKCs (ζ, λ/ι) that are not dependent on either DAG or calcium.

Opposite roles have been described for PKC isoenzymes in tumor promotion; PKCε has been shown to act as a transforming oncogene and to confer tumorigenic phenotype in nude mice [4]. By contrast, the suppression of PKCδ expression or down-regulation of its activity is believed to favor a transformed phenotype [5]. In particular, two PKC isoenzymes play specific roles in cell survival and apoptosis: PKCα promotes EGF-transforming activity [6] and is generally described as anti-apoptotic [7], whereas PKCδ has anti-proliferative effects [8], promoting cell differentiation [9] and mediating pro-apoptotic events [10]. The tumor suppressor ability of PKCδ likely involves the Ras/Raf/MEK/MAP kinase-signaling pathway [11].

We have previously demonstrated that cell death, triggered by L-buthionine-S,R-sulfoximine (BSO), a glutathione (GSH)-depleting agent, is mediated by PKCδ activation and reactive oxygen species (ROS) overproduction [12].

In the present study, we investigated whether PKCδ could sensitize neuroblastoma (NB) cell lines to apoptosis. Our results indicate that overexpression of PKCδ in GSH-depleted cells leads to ROS overproduction that is responsible for DNA oxidative damage and apoptosis, two events efficiently prevented by diphenyleneiodonium (DPI), a flavoprotein widely used to inhibit NADPH oxidase. The crucial role of PKCδ is also observed in NB cells exposed to low doses of etoposide, a major anti-tumor agent used for the treatment of NB [13]. Altogether, our data imply that nuclear translocation of the functionally-active full-length PKCδ is an early and important step necessary to prime the apoptotic pathway in response to cytotoxic drugs.

## Materials and Methods

[γ^32^P] ATP was from Perkin Elmer Life and Analytical Sciences (Shelton, CT, USA). Etoposide was from Calbiochem (Merck KGaA, Darmstadt, Germany). All other chemicals were from Sigma-Aldrich (St. Louis, Mo, USA).

### Cell cultures and transfections

Human NB cell lines ACN, GI-MEN, SH-SY-5Y and SK-N-BE-2C were purchased from the Bank of Biological Material Interlab Cell Line Collection, Advanced Biotechnology Center, Genoa, Italy. Cells were cultured in RPMI 1640 (Euroclone s.p.a, Pavia, Italy) supplemented with 10% fetal bovine serum (FBS; Euroclone), 2 mM L-glutamine, 1% penicillin/streptomycin, 1% sodium pyruvate and 1% of aminoacid solution.

PKCδ and Dominant-Negative PKCδ cDNA (K376M) [14], both cloned into pEGFP-N1 vector (Clontech Laboratories Inc, Mountain View, CA, USA), were kindly provided by Dr. C. Larsson (University of Malmö, Malmö, Sweden) and Prof. P. Parker (Cancer Research UK, London, UK), respectively. The coding sequence of the EGFP gene was removed by cutting the PKCδ plasmid with EcoRI and NotI restriction enzymes (Roche Diagnostics, GmbH, Mannheim, Germany). The integrity of the resulting PKCδ construct was confirmed by sequencing. Transient transfections were performed using Lipofectamine 2000 (Invitrogen Srl, S. Giuliano Milanese, Italy) at 2.5 µl/µg DNA.

### siRNA transfection

Gene silencing by small interfering RNA (siRNA) was carried out by transfecting cells with a non-targeting pool “NoT”, used as a negative control, and with SMARTpool siRNA oligonucleotides, used to interfere with human PKCδ expression (ON-TARGETEDplus, Dharmacon, Lafayette, CO, USA) employing INTERFERin™ (Polyplus Transfection) according to the manufacturer's instructions. The cells were then transfected with oligonucleotides at a final concentration of 5 nM. After 48 h, transfection reagents were washed out and the cells were stimulated for 24 h with 1 mM BSO or 0.07 µM etoposide.

### Apoptosis/necrosis detection assays

For the assessment of apoptosis and necrosis, cells were analysed as previously described [15]. Briefly, after treatment cells were incubated with 0.5 µg/ml FITC-labelled recombinant Annexin V and 0.5 µg/ml propidium iodide (PI; BioVision, Mountain View, CA, USA). Cells were visualised and counted (4 fields of 200-400 cells) by fluorescence microscopy using a Leica DMIRB microscope with a dual filter set for FITC and rhodamine. Images were acquired with a Leica DCF320 camera. Cell death was expressed as a percentage of Annexin-V and PI positive cells.

### Caspase-3 activity assay

The activity of caspase-3 was measured spectrophotometrically using a 96-well microplate kit (Caspase-3 Cellular Activity Assay Kit, Calbiochem). Briefly, 10 µl of cell extract and 40 µl assay buffer (100 mM NaCl, 50 mM HEPES, 10 mM DTT, 1 mM EDTA, 10% glycerol, 0.1% CHAPS, cholamidopropyl-dimethylammonio-1-propanesulfonate, pH 7.4) were added to the wells and the microplate was equilibrated at 37°C. The reaction was initiated by adding the caspase-3 colorimetric tetrapeptide substrate N-acetyl-Asp-Glu-Val-Asp-p-nitroanilide (DEVD-pNa, 200 µM final concentration). Absorbance was recorded at 7 min intervals for 3 h at 405 nm using a microplate reader (EL-808, BIO-TEK Instruments Inc., Winooski, VT, USA). All samples were analyzed in duplicates and purified activated caspase-3 was included on each microplate as a positive control.

Specific activity of each sample was evaluated in respect to the total protein content and was reported as a percentage relative to the untreated control.

### MTT Assay

Cell viability was determined using the dimethylthiazolyl-2-5-diphenyltetrazolium bromide (MTT, Sigma) staining. In brief, cells were seeded into 6 well-plates (Corning, Corning Incorporated, NY, USA), transfected with the empty vector (EV) or PKCδ plasmid and then treated with 1 mM BSO for 24 h. Next, cells were incubated with 0.5 mg/ml MTT for 3 h at 37°C. After incubation, the supernatant was discarded, insoluble formazan precipitates were dissolved in 0.1 ml HCl (0.1 N in isopropanol) and the absorbance at 570 nm was recorded using a microplate reader (EL-808, BIO-TEK Instruments Inc.). Each experiment was performed in triplicate. Values from each treatment were calculated as a percentage relative to the untreated control.

### Immunoblot analysis

Immunoblotting was carried out according to standard methods [16], using the following antibodies: polyclonal rabbit anti-human PKCδ (Cell Signaling Technology Inc., Danvers, MA, USA and Santa Cruz Biotechnology, Santa Cruz, CA, USA), anti phospho-PKCδ (Thr505) (Cell Signaling), monoclonal mouse anti-PKCα and polyclonal rabbit anti-PKCε (Upstate, Lake Placid, NY, USA), monoclonal mouse anti-β actin (Sigma) and, finally, anti-mouse and anti-rabbit secondary antibodies coupled with horseradish-peroxidase (Amersham International, Buckinghamshire, UK). Proteins were visualized with an enzyme-linked chemiluminescence detection kit according to the manufacturer's instructions (Amersham). Chemiluminescence was monitored by exposure to film and the signals were analyzed under non-saturating conditions with an image densitometer and Quantity One software (Bio-Rad Laboratories, Hercules, CA, USA).

### PKC activity assay

After treatment, PKCδ was immunoprecipitated with polyclonal rabbit anti-human PKCδ antibody (Cell Signaling and Santa Cruz) and protein A/G-sepharose (Santa Cruz) from 50 µg of total cell extracts. Activity assay was performed as previously described [15], using H1 Histone as a substrate [17]. The relative intensity of the phosphorylated substrate was quantified by densitometric analysis.

### Detection of ROS

Detection of ROS was performed as described in a previous work [15]. Briefly, after treatment, cells were incubated with 20 µM 2′-7′ dichlorofluorescein-diacetate (DCFH-DA; Sigma) and the accumulation of dichlorofluorescein (DCF) was measured by the increase in fluorescence at 530 nm, exciting the samples at 485 nm [18]. The cells were observed and counted (4 fields of 200–400 cells) by fluorescence microscopy using a Leica DMIRB microscope with a standard set of filters for fluorescein. The images were acquired with a Leica DCF320 camera.

### Single cell gel electrophoresis (Comet assay)

Comet assay detects DNA damage at the level of single cells. Formamidopyrimidine DNA glycosylase (fpg)-modified comet assay [19] was used to evaluate DNA damage of oxidative nature. This test utilizes the fpg enzyme, a glycosylase that recognizes and specifically removes the oxidized bases from DNA, principally 8-OH deoxyguanosine (8-OHdG), producing apurinic sites converted in breaks by the associated AP-endonuclease activity. After treatment, cells were trypsinized and the cell suspensions (1.5×10^4^ cells) were mixed with low melting point agarose and spread on slides covered with a thin layer of normal melting point agarose. Then, slides were washed and incubated with fpg (1 µg/ml) in the dark for 30 min at 37°C. After the electrophoresis at 300 mA for 40 min, the slides were treated with absolute ethanol and then stained with ethidium bromide. Comet capture was performed using a Leica DIMRB fluorescence microscope (excitation filter of 515–560 nm and a barrier filter of 590 nm) and the images were acquired with a Leica DCF320 camera. DNA strand breaks were expressed as tail moment data calculated by measuring the resulting comets by TriTek CometScore™ Freeware image analysis software (TriTek Corporation). At least 4 fields of 25 randomly selected cells pooled from three independent experiments were analyzed.

### Detection of PKCδ translocation by confocal microscopy

Cells were fixed and permeabilized with cold (−20°C) methanol immediately before being processed for immunofluorescence. Non-specific antibody binding was blocked by a 30 min incubation with 5% (v/v) fetal calf serum. Cells were then treated with 2 µg/ml rabbit anti-human PKCδ (Santa Cruz), followed by an Alexa Fluor488 anti-rabbit secondary antibody (Invitrogen). Nuclei were identified with PI staining. Mitochondria were labelled with MitoTraker Deep Red FM (Invitrogen) according to the manufacturer's instructions. Images were collected by confocal microscopy using a Leica TCS SL2 instrument (Leica Wetzlar, Germany), equipped with argon/He-Ne laser sources and an HCX PL APO CS 63.0×1.40 oil objective. Excitation and emission wavelengths were 488 and 522 nm respectively for Alexa-labelled antibody, 543 and 605 nm for PI staining and 633 and 662 nm for MitoTraker Deep Red FM labelling. Sequential acquisition was performed to avoid cross-talk between color channels. Nuclear translocation was analyzed through ImageJ 1.34f software (Wayne Rasband, National Institutes of Health, Bethesda, MD, USA).

### Data analysis

Results were expressed as mean ± SD from at least three independent experiments. The statistical significance of parametric differences among sets of experimental data was evaluated by one-way ANOVA and Dunnett's test for multiple comparisons.

## Results

### Overexpression of PKCδ enhances ROS production in NB cell lines

**Figure 1:**
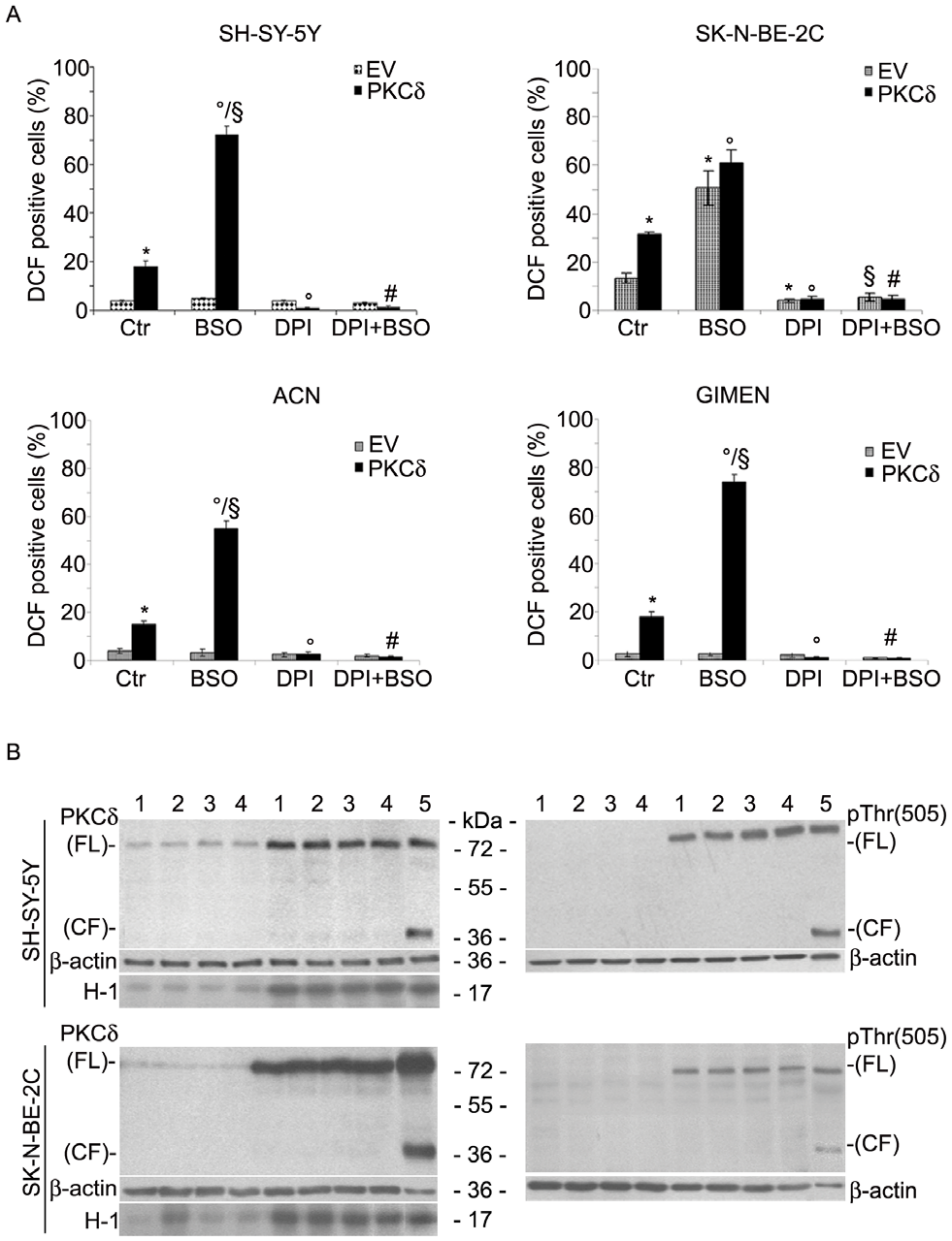
PKCδ overexpression increases ROS production in untreated and BSO-treated NB cells. **A**, SH-SY-5Y, ACN, GI-MEN and SK-N-BE-2C cells were transfected with empty vector (EV) or PKCδ plasmid. 24 h after transfection, cells were treated with 1 mM BSO for 24 h. Where indicated, cells were pre-treated with 25 nM DPI for 30 min. ROS analysis was performed as described in Materials and Methods. Histograms summarize quantitative data of means ± SD of five independent experiments. * p<0.01 vs. EV transfected cells; ° p<0.01 vs. PKCδ transfected cells;^§^ p<0.01 vs. EV transfected cells +BSO; ^#^ p<0.01 vs PKCδ transfected cells +BSO. **B**, Immunoblot of SH-SY-5Y and SK-N-BE-2C protein extracts (10 µg/lane) was performed with anti-PKCδ (left panels) and anti-phosphoThr (505) PKCδ (right panels) antibodies. Lanes 1 show the protein extracts from untreated cells, lanes 2 from BSO-treated cells, lanes 3 from DPI-treated cells and lanes 4 from DPI+BSO-treated cells. Protein extracts from SH-SY-5Y cells transfected with PKCδ and exposed to 100 µM H_2_O_2_ (4 h) were used as positive control (lane 5). β-actin signals represent the immunoblot loading control. PKCδ activity was measured by phosphorylation of H1 histone, utilized as a substrate (see Materials and Methods). The immunoblots shown are representative of five independent experiments.

In the present study, three human NB cell lines, without MYCN amplification (ACN, GI-MEN, SH-SY-5Y) and a MYCN-amplified cell line (SK-N-BE-2C) were transiently transfected with an expression vector encoding PKCδ and incubated with 1 mM BSO for 24 h. Transfection efficiency (percentage of pEGFP-N1-PKCδ positive cells) was about 80–85% in SH-SY-5Y, ACN and GI-MEN while it was 40–45% in SK-N-BE-2C cells. ROS production was analyzed by fluorescence microscopy as described in Materials and Methods and measured as a percentage of DCF positive cells. SH-SY-5Y, ACN and GI-MEN cells, transfected with the EV, did not generate ROS under any treatment conditions, while the same cell lines, transfected with PKCδ expressing vector, showed a 15–18% of DCF positivity that increased to 55–74% after BSO treatment (24 h). In SK-N-BE-2C cells transfected with EV, the DCF positivity was already 12% and increased 4-fold after BSO incubation (Figure 1A). However, when these cells were transfected with PKCδ plasmid, the percentage of DCF-positive cells was 30%, doubling to 60% after BSO exposure. Inhibition of NADPH oxidase by diphenyleneiodonium chloride (DPI), either alone or associated with BSO, was capable of totally suppressing ROS production (Figure 1A).

### PKCδ protein levels and enzymatic activities are not influenced by BSO and DPI

NB cells were analyzed after transfection with PKCδ expressing vector in order to test the kinase expression pattern. Since the results were similar in all the cell lines tested (data not shown), we have selected SH-SY-5Y cells as the prototype of MYCN-non-amplified cells, and SK-N-BE-2C as the model of MYCN-amplified cells. In both cell lines that overexpress PKCδ, the profile of other PKC isoforms, such as α and ε, were found to be unaltered (data not shown). As reported in Figure 1B, treatment of SH-SY-5Y and SK-N-BE-2C cells with BSO and/or DPI did not change the expression level of PKCδ, the basal content of which was low in both cell lines. PKCδ-overexpressing cells showed Thr505 phosphorylation, a marker of the enzyme activation [9] and, after BSO and/or DPI incubations, the phosphorylation status of the PKCδ residue was not significantly modified.

Since it is known that PKCδ may be activated by proteolytic cleavage [20], [21], the presence of the 40 kDa catalytic fragment was investigated in both cell lines under all conditions. Immunoblot analysis showed that neither BSO nor DPI, alone or together, induced the proteolytic cleavage of PKCδ that was, however, observed in the positive control, represented by PKCδ-transfected SH-SY-5Y cells exposed to 100 µM H_2_O_2_ (Figure 1B).

BSO stimulated the PKCδ enzymatic activity of SK-N-BE-2C cells 3-fold, whereas DPI pre-treatment exerted an efficient preventive effect (Figure 1B). In PKCδ-overexpressing cells, the kinase activity was not modified by BSO and/or DPI exposure (Figure 1B).

In SH-SY-5Y cells, BSO treatment, alone or associated with DPI, did not influence the activity of endogenous and exogenous PKCδ (Figure 1B).

### PKCδ overexpression is crucial for BSO-mediated apoptotic death

**Figure 2:**
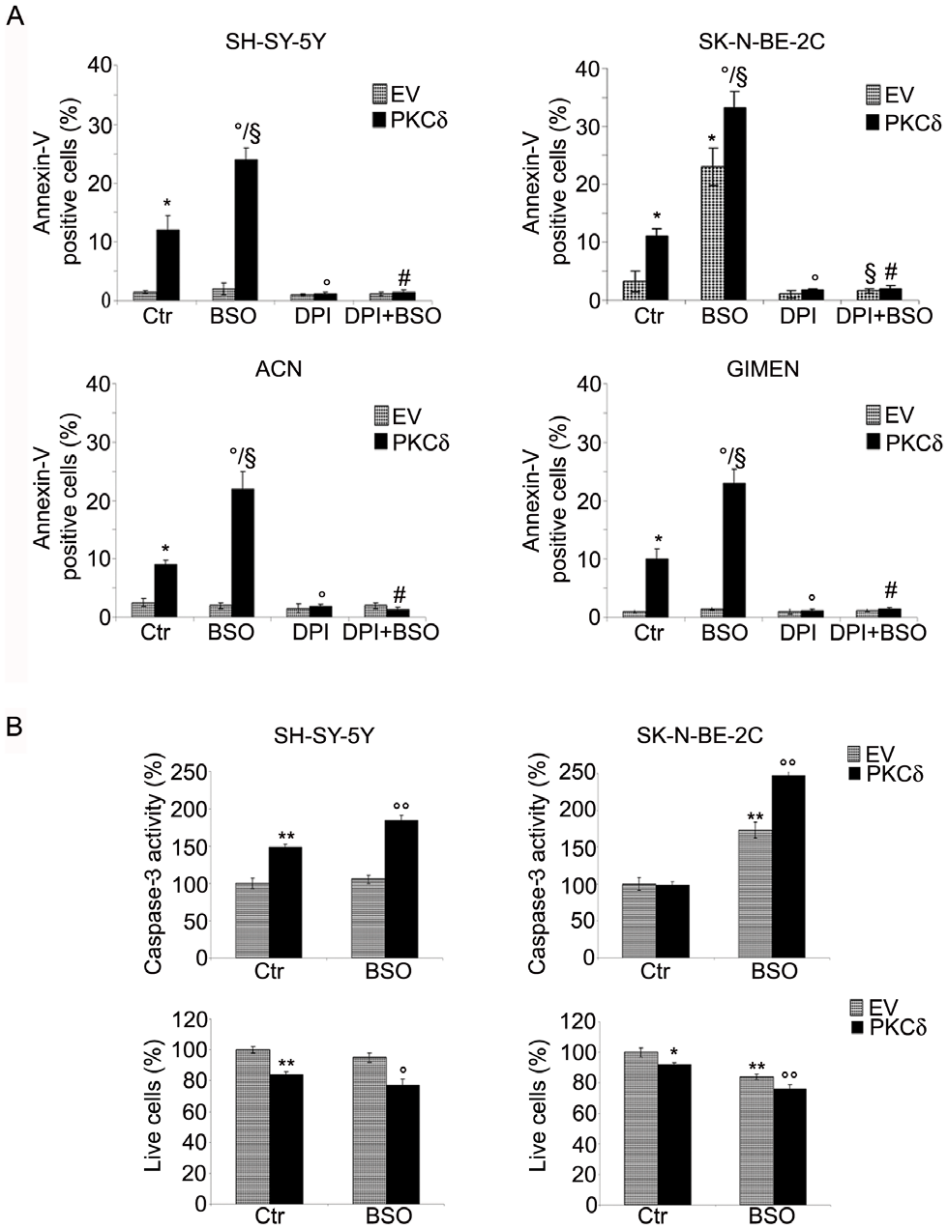
Overexpression of PKCδ sensitizes NB cells to BSO-induced apoptosis. **A**, After transfections and treatments (performed as in Fig. 1), apoptosis was analyzed by fluorescence microscopy using Annexin-V/PI assay (see Materials and Methods). Histograms summarize quantitative data of means ± SD of five independent experiments. * p<0.01 vs. EV transfected cells; ° p<0.01 vs. PKCδ transfected cells; ^§^ p<0.01 vs. EV transfected cells +BSO; ^#^ p<0.01 vs PKCδ transfected cells +BSO. **B,** Caspase-3 activity was analyzed by Caspase-3 Cellular Activity Assay Kit (see Materials and Methods) in SH-SY-5Y (left panel) and SK-N-BE-2C cells (right panel). The percentage of living cells was determined by MTT analyses. Histograms summarize quantitative data of means ± SD of three independent experiments. * p<0.05 vs. EV transfected cells; ** p<0.01 vs. EV transfected cells; ° p<0.05 vs. PKCδ transfected cells; °° p<0.01 vs. PKCδ transfected cells.

Cell viability was tested by analyzing the rate of Annexin-V/propidium iodide positive cells. As shown in Figure 2A, treatment with BSO did not modify the viability of SH-SY-5Y, ACN and GI-MEN cells, but was able to induce apoptosis in SK-N-BE-2C cells, evaluated as 22% of Annexin-V positive cells. The transient transfection with PKCδ itself induced a 10–14% of Annexin-V positivity in all cell lines tested. Moreover, after BSO exposure, a 20–24% of Annexin-V positivity was recorded in all PKCδ-transfected cells except for SK-N-BE-2C, in which the percentage of apoptosis incremented, and reached a 35% value.

The blocking of ROS production by DPI protected all PKCδ-overexpressing cells and also EV-transfected SK-N-BE-2C cells from apoptosis. Under all conditions, PI positive cells were not detected (data not shown), demonstrating the absence of necrotic events.

To understand the mechanism of the enhanced apoptotic process triggered by BSO exposure and PKCδ overexpression, caspase-3 activity and MTT analyses were carried out. As shown in Figure 2B, BSO treatment did not affect the activity of caspase-3 measured in EV-transfected SH-SY-5Y cells. Conversely, when the same cells were transfected with PKCδ, the activity of caspase-3 increased by 50%, when compared to EV-transfected cells, and further increased by 33% after BSO exposure. In SK-N-BE-2C cells, the caspase-3 activity was comparable in EV- and in PKCδ-transfected cells. However, BSO treatment of EV- and PKCδ-transfected cells led to a significant increase in caspase-3 activity by 70% and 146% respectively.

These results were further supported by a MTT assay (Figure 2B) which showed that the viability of SH-SY-5Y cells overexpressing PKCδ was reduced by 28% after BSO incubation. This latter treatment was seen to lower the viability of SK-N-BE-2C cells by 16% and by 24% after PKCδ overexpression.

### PKCδ overexpression leads to oxidative DNA damage

DNA damage was analyzed under all experimental conditions using comet assay. As shown in Table 1, the DNA from EV-transfected SH-SY-5Y, ACN and GI-MEN cells was found not to be damaged neither after BSO treatment nor DPI pre-treatment, whereas in SK-N-BE-2C, a marked DNA damage was recorded after BSO exposure, with DPI preventing the genotoxic effect. Apparently, PKCδ overexpression, in all cell lines tested, did not affect the integrity of DNA, which was impaired after BSO and protected by DPI. In order to verify whether the observed DNA damage was of an oxidative nature, the same analysis was performed in the presence of fpg (see Materials and Methods). In fact, this approach revealed that PKCδ overexpression *per se* was responsible for DNA oxidation that became more pronounced after BSO treatment and was efficiently prevented by DPI (Table 1).

**Table 1:** PKCδ overexpression is accompanied by DNA damage.

		SH-SY-5Y		SK-N-BE-2C	
		Standard (A.U.)	fpg (A.U.)	Standard (A.U.)	fpg (A.U.)
**CTR**	**EV**	0.0006±0.0001	0.0004±0.0001	0.0008±0.0001	0.0006±0.0001
	**PKCδ**	0.0006±0.0001	12.111±4.632**,°°	0.0009±0.0001	7.321±1.632**,°°
**BSO**	**EV**	0.0005±0.0001	0.0005±0.0001	10.532±1.326**	12.569±1.315**
	**PKCδ**	20.4718±3.9458§§	17.5194±3.9908§§	20.4718±3.9458§	17.5194±3.9908§
**DPI**	**EV**	0.0004±0.0001	0.0007±0.0002	0.0008±0.0002	0.0007±0.0004
	**PKCδ**	0.0006±0.0001	0.0004±0.0001••	0.0009±0.0002	0.0007±0.0002••
**DPI+BSO**	**EV**	0.0003±0.0001	0.0005±0.0001	0.0004±0.0001§§	0.0004±0.0001§§
	**PKCδ**	0.0005±0.0001◊◊	0.0004±0.0001◊◊	0.0006±0.0001◊◊	0.0005±0.0001◊◊

		ACN		GI-MEN	
		Standard (A.U.)	fpg (A.U.)	Standard (A.U.)	fpg (A.U.)
**CTR**	**EV**	0.0023±0.0005	0.0023±0.0004	0.0009±0.0003	0.0002±0.0001
	**PKCδ**	0.0014±0.0002	3.047±1.0985**,°°	0.0005±0.0002	8.0740±2.7906**,°°
**BSO**	**EV**	0.0022±0.0005	0.0015±0.0003	0.0007±0.0001	0.0005±0.0001
	**PKCδ**	17.7651±5.4857§§	12.1395±5.1406§§	20.4955±4.5755§§	12.3684±4.8728§§
**DPI**	**EV**	0.0012±0.0002	0.0018±0.0003	0.0005±0.0001	0.00049±0.0001
	**PKCδ**	0.0011±0.0003	0.0007±0.0005••	0.0004±0.0001	0.0005±0.0001••
**DPI+BSO**	**EV**	0.0017±0.0011	0.0012±0.0005	0.0005±0.0001	0.0004±0.0001
	**PKCδ**	0.0006±0.0001◊◊	0.0007±0.0003◊◊	0.0004±0.0001◊◊	0.0003±0.0001◊◊

NB cells, transiently transfected with empty vector (EV) or PKCδ plasmid, were treated with 1 mM BSO for 24 h. Where indicated, cells were pre-treated with 25 nM DPI for 30 min. DNA fragmentation (standard) and oxidation (fpg) were evaluated by comet test. The reported values derive from tail moment analyses.

**p<0.01 vs EV CTR/fpg;

°°*p*<0.01 vs PKCδ CTR standard;

§ *p*<0.05 vs EV + BSO;

§§ *p*<0.01 vs EV + BSO;

•• *p*<0.01 vs PKCδ CTR fpg;

◊◊ *p*<0.01 vs PKCδ+BSO.

### The susceptibility to etoposide or BSO of PKCδ overexpressing cells is prevented by rottlerin

**Figure 3:**
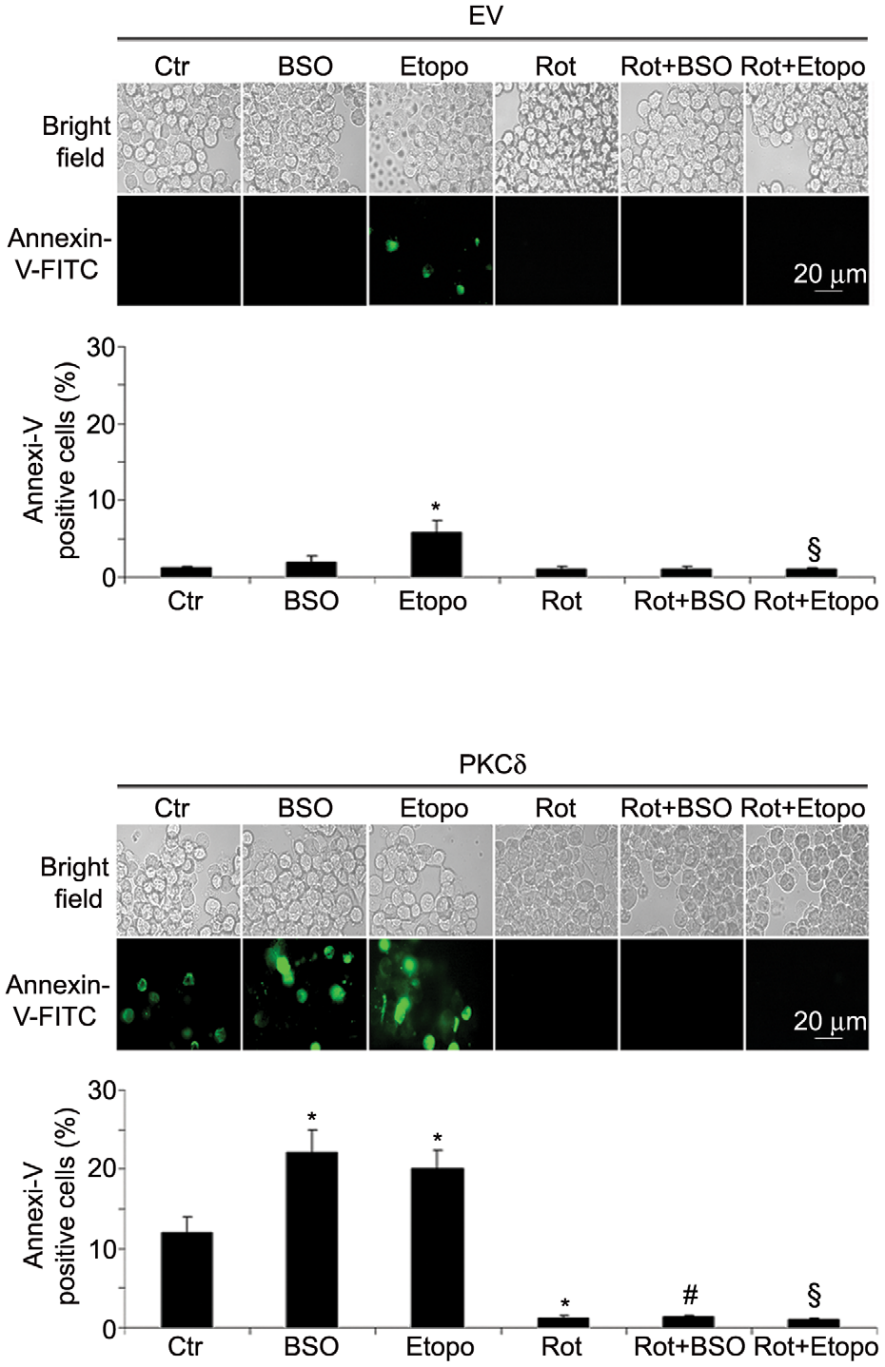
PKCδ overexpression increases the pro-apoptotic effects of etoposide. SH-SY-5Y cells, transiently transfected with empty vector (EV) or PKCδ, were treated with etoposide (0.07 µM) or BSO (1 mM) for 24 h. Where indicated, cells were pre-treated with rottlerin (3 µM) for 30 min. After treatments, cells were processed for Annexin V assay. Data is expressed as means ± SD of three independent experiments. * p<0.01 vs untreated EV or PKCδ-transfected cells; ^§^ p<0.01 vs. EV or PKCδ-transfected cells treated with etoposide; ^#^ p<0.01 vs PKCδ-transfected cells treated with BSO.

Considering the role of PKCδ in the induction of oxidative cell death, we then investigated whether the kinase overexpression could increase the susceptibility of NB cells to etoposide, a drug widely used in the therapy of neuroblastoma. For this purpose, wild type SH-SY-5Y cells were exposed for 24 h to a range of etoposide concentrations (0.07, 0.1, 0.4, 0.8, 1.25 µM) compatible with those used in clinical practice [22]. The lowest etoposide dose (0.07 µM) caused a slight but significant increase in the rate of apoptosis without inducing necrosis. Conversely, higher etoposide doses had a marked necrotic effect (data not shown). Next, we evaluated the effect of 0.07 µM etoposide on the DNA of EV-transfected SH-SY-5Y cells using comet assay. As shown in Table 2, etoposide alone caused oxidative damage to DNA which was suppressed by rottlerin, an inhibitor of novel PKCs [23]. When the cells were transfected with PKCδ, exposure to either etoposide (0.07 µM) or BSO (1 mM) induced DNA fragmentation and oxidation. Moreover, under the same conditions, the number of apoptotic cells was significantly increased (20-22% vs 12% of untreated PKCδ-overexpressing cells; Figure 3). Rottlerin pre-incubation prevented apoptosis (Figure 3) and oxidative damage to DNA (Table 2), both having been triggered by etoposide or BSO, and in addition suppressed the effects caused by the PKCδ overexpression itself.

**Table 2:** PKCδ overexpression amplifies the genotoxic effects of etoposide.

		SH-SY-5Y	
		Standard (A.U.)	fpg (A.U.)
**CTR**	**EV**	0.0006±0.0001	0.0004±0.0001
	**PKC δ**	0.0006±0.0001	12.111±4.632**,°°
**BSO**	**EV**	0.0005±0.0001	0.0005±0.0001
	**PKC δ**	20.4718±3.9458§§	17.5194±3.9908§§
**ETOPO**	**EV**	0.0004±0.0001	9.0121±3.5361**,§§
	**PKC δ**	22.0825±4.1479§§	16.1373±3.5423§§
**ROT**	**EV**	0.0004±0.0002	0.0004±0.0001
	**PKC δ**	0.0005±0.0002	0.0003±0.0001••
**ROT+BSO**	**EV**	0.0004±0.0001	0.0003±0.0001
	**PKC δ**	0.0004±0.0001◊◊	0.0003±0.0002◊◊
**ROT+ETOPO**	**EV**	0.0005±0.0003	0.0002±0.0001##
	**PKC δ**	0.0003±0.0002◊◊	0.0003±0.0002◊◊

SH-SY-5Y cells, transiently transfected with empty vector (EV) or PKCδ plasmid, were treated with 0.07 µM etoposide or 1 mM BSO for 24 h. Where indicated, cells were pre-treated with rottlerin for 30 min. DNA fragmentation (standard) and oxidation (fpg) were evaluated by comet test. The reported values derive from tail moment analyses.

**p<0.01 vs EV CTR/fpg;

°°*p*<0.01 vs PKCδ CTR standard;

§§ *p*<0.01 vs EV + BSO/ETOPO;

## *p*<0.01 vs EV ETOPO fpg;

•• *p*<0.01 vs PKCδ CTR fpg;

◊◊ *p*<0.01 vs PKCδ+BSO/ETOPO.

### PKCδ is required to induce the oxidative death and the genotoxic effects triggered by etoposide and BSO

**Figure 4:**
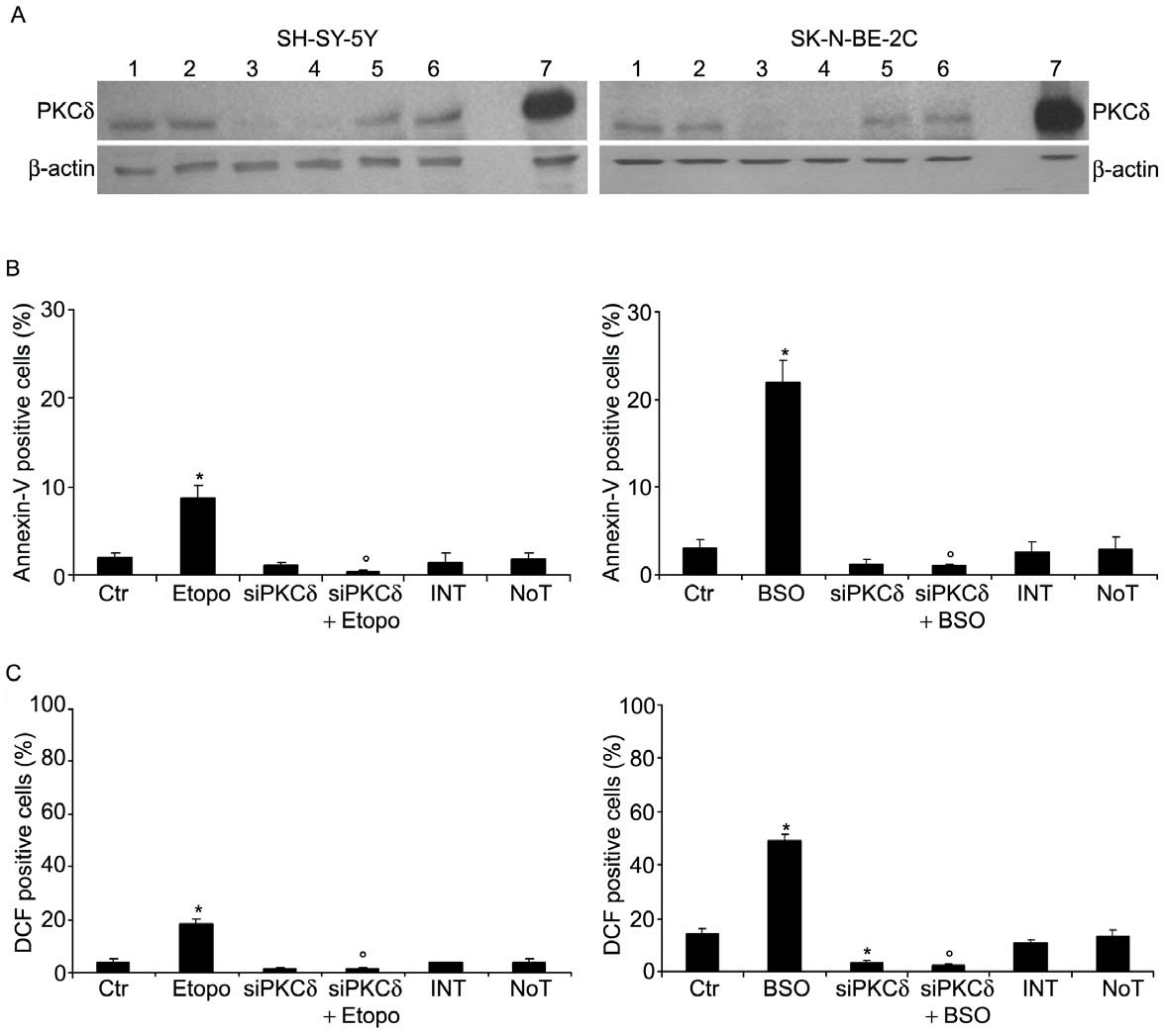
PKCδ silencing prevents apoptosis and ROS over-production triggered by etoposide and BSO. Down-regulation of endogenous PKCδ was obtained using the technique of RNA silencing, as described in Materials and Methods. After 48 h transfection, SH-SY-5Y (left panels) and SK-N-BE-2C cells (right panels) were stimulated for 24 h with 0.07 µM etoposide or 1 mM BSO. **A**, Immunoblots of SH-SY-5Y and SK-N-BE-2C protein extracts (20 µg/lane) were performed with anti-PKCδ and anti-β actin antibodies. Lanes 1 show the protein extracts from untreated cells, lanes 2 from etoposide/BSO-treated cells, lanes 3 from untreated cells transfected with PKCδ siRNA, lanes 4 from cells transfected with PKCδ siRNA and then treated with etoposide/BSO, lanes 5 from cells transfected with siCONTROL RNA (NoT), lanes 6 from cells exposed to the agent used for silencing (INT, INTERFERin^TM^) and lanes 7 from the positive control (as detailed above). The immunoblots shown are representative of five independent experiments. **B,** After silencing and treatments, apoptosis was analyzed by fluorescence microscopy using Annexin-V/PI assay (see Materials and Methods). **C;** ROS analysis was performed as described in Materials and Methods. Histograms summarize quantitative data of means ± SD of five independent experiments. * p<0.01 vs. untreated cells; ° p<0.01 vs. cells treated with etoposide/BSO.

To confirm the direct involvement of PKCδ in BSO- and etoposide-induced effects, SK-N-BE-2C and SH-SY-5Y cells, after transfection with both non-targeting siCONTROL RNA and PKCδ-specific siRNA, were respectively treated with BSO or etoposide for 24 h. As shown in Figure 4A, in both cell lines, PKCδ levels were markedly reduced (90±7.2%) by the specific silencing and the treatments with etoposide or BSO did not change the protein levels. Moreover, the treatment with non-targeting siCONTROL RNA (NoT) did not affect the amount of PKCδ (Figure 4A).

As shown for pre-treatment with rottlerin, silencing of PKCδ efficiently prevented apoptosis (Figure 4B), ROS overproduction (Figure 4C) and also oxidative DNA damage (Table 3) observed in etoposide-treated SH-SY-5Y cells. Similarly, silencing of PKCδ was able to markedly protect SK-N-BE-2C cells from BSO-induced effects (Figure 4B, C and Table 3).

**Table 3:** Silencing of PKCδ prevents the genotoxic effects triggered by etoposide and BSO in SH-SY-5Y and in SK-N-BE-2C cells, respectively.

		SH-SY-5Y		SK-N-BE-2C	
		standard (A.U.)	fpg (A.U.)	Standard (A.U.)	fpg (A.U.)
**CTR**	**NoT**	0.0006±0.0001	0.0004±0.0001	0.0008±0.0001	0.0006±0.0001
	**siPKCδ**	0.0006±0.0001	0.0007±0.0001	0.0009±0.0001	0.0008±0.0001
**ETOPO**	**NoT**	0.0004±0.0001	8.352±2.8754**,§§	-	-
	**siPKCδ**	0.0006±0.0001	0.0004±0.0001##	-	-
**BSO**	**NoT**	-	-	11.325±2.184♦♦	13.215±1.969**
	**siPKCδ**	-	-	0.0007±0.0001§§	0.0006±0.0001##

NB cells, silenced for PKCδ, were treated with 0.07 µM etoposide (SH-SY-5Y) and 1 mM BSO (SK-N-BE-2C) for 24 h. DNA fragmentation (standard) and oxidation (fpg) were evaluated by comet test. The reported values derive from tail moment analyses.

**p<0.01 vs NoT CTR fpg;

♦♦ *p*<0.01 vs NoT CTR standard;

§§ *p*<0.01 vs NoT + ETOPO/BSO standard,

## *p*<0.01 vs NoT ETOPO/BSO fpg.

### BSO and etoposide induce early translocation of PKCδ to the nucleus

**Figure 5:**
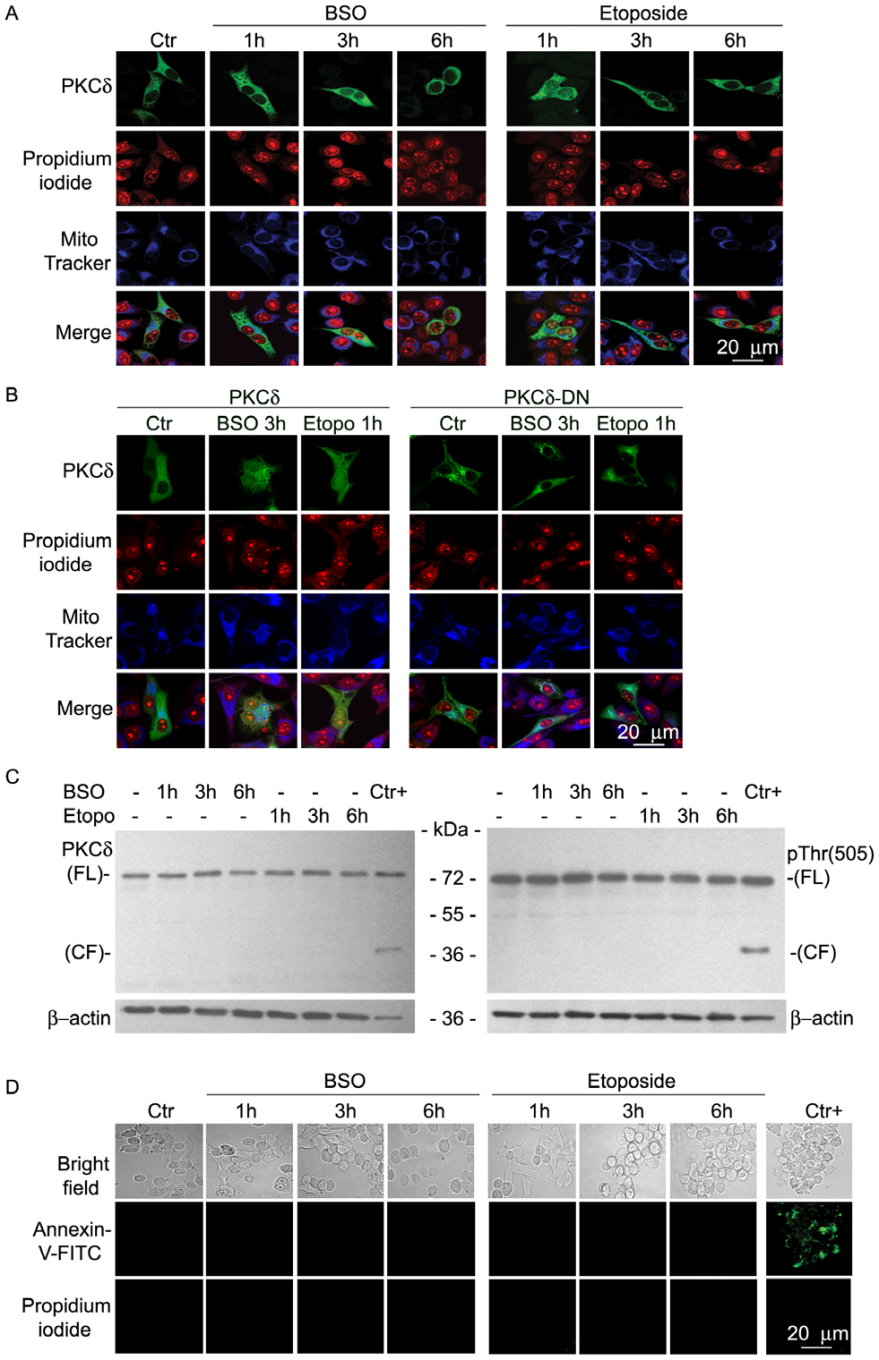
PKCδ subcellular localization and cell viability in NB cells treated with BSO or etoposide. **A,** Confocal microscopy images of SH-SY-5Y cells transiently transfected with PKCδ and treated with etoposide (0.07 µM) or BSO (1 mM) for 1, 3 and 6 h. After treatment, cells were fixed, permeabilized and stained with anti-PKCδ antibody (green), propidium iodide (red) and MitoTraker (blue). **B**, Confocal microscopy images of SH-SY-5Y cells transfected with pEGFP-PKCδ/pEGFP-PKCδ-DN plasmid and treated with 1 mM BSO (3 h) or 0.07 µM etoposide (1 h). After treatment, cells were fixed and labelled as detailed above. **C,** Immunoblots of PKCδ–transfected SH-SY-5Y protein extracts (10 µg/lane) were performed with isoform specific anti-PKCδ and anti phospho-PKCδ (Thr505) antibody (see Materials and Methods). **D**, Fluorescence microscopy images of PKCδ–transfected cells, treated as indicated and labelled with FITC-conjugated Annexin V/PI. The figures shown are representative of experiments performed in triplicate.

In unstimulated cells, PKCδ resides in the cytosol and, upon treatment with the appropriate agonists, translocates to membrane compartments [24]. Therefore, we investigated, by confocal microscopy, the subcellular localization of PKCδ soon after BSO or etoposide treatment. As shown in Figure 5A, BSO (3 h) and etoposide (1 h) induced the partial translocation of PKCδ to the nucleus, as indicated by the overlapping of green fluorescence (PKCδ) with the red nuclear counterstain. It should be noted that PKCδ did not translocate to mitochondria, as demonstrated by the absence of the overlapping of green fluorescence (PKCδ) with MitoTraker Deep Red FM labelling. To investigate whether the nuclear translocation is dependent on the activation of PKCδ, SH-SY-5Y cells were transfected, in parallel experiments, with PKCδ and with Dominant Negative PKCδ (PKCδ-DN) plasmid, the resulting overexpressing cells then being treated either with BSO or etoposide. Since the results obtained, by labelling cells with the PKCδ antibody or by observing the localization of PKCδ-GFP, were similar, the comparison between localization of PKCδ and PKCδ-DN, at the time in which the kinase moves to the nucleus, was done using both plasmids carrying GFP. As shown in Figure 5B, both PKCδ and PKCδ-DN were distributed into the cytosol of untreated cells. Surprisingly, after BSO (3 h) and etoposide (1 h) treatment, PKCδ partially translocated to the nucleus (21±10%) whereas PKCδ-DN remained localized in the cytosol (Figure 5B). Immunoblot analysis excluded the formation of PKCδ catalytic fragments and showed that the transfected PKCδ was phosphorylated at the Thr505 residue (pThr505) (Figure 5C). Interestingly, after incubation (1, 3 and 6 h) with BSO or etoposide, PKCδ-overexpressing cells were viable (Figure 5D) and DNA was neither fragmented nor oxidized (data not shown).

In order to investigate whether the observed genotoxic action of PKCδ was a direct consequence of the sole increase in its protein levels or if it was due to its activity, SH-SY-5Y cells, transfected with PKCδ-DN and exposed to either BSO or etoposide for 24 h, were analyzed in terms of DNA damage. Standard comet assay demonstrated that the transfection with the PKCδ-DN had not induced DNA damage, as demonstrated by analyzing the tail moments of the comets (0.0005±0.0001 in untreated PKCδ-DN cells; 0.0006±0.0001 and 0.0004±0.0001 in BSO or etoposide exposed cells, respectively). The fpg-modified comet assay revealed that the transfection with PKCδ-DN induced a pattern of DNA damage similar to that observed in EV-transfected cells (0.0005±0.0001 in untreated and in BSO-treated PKCδ-DN cells and 8.532±4.658 in etoposide exposed cells).

These data suggest that the functionally active PKCδ is able to sensitize NB cells to BSO and etoposide by inducing ROS overproduction and DNA damage.

## Discussion

Pharmacological GSH depletion by BSO highly sensitizes tumor cells to apoptosis induced by chemotherapeutic agents [25], [26]. Recently, it has been found that GSH deficiency is associated with elevated levels of 8-OHdG and DNA single strand-breaks in cell cultures [27]. Moreover, the lack of GSH induces DNA deletions in mice [28] and DNA oxidative modifications in rabbits [29].

Our previous studies have shown that MYCN-non-amplified NB cell lines are more resistant to apoptosis induced by BSO, compared with MYCN amplified cells [30]. In recent literature, it has been reported that human NB cells with MYCN amplification are selectively resistant to oxidative stress by transcriptionally up-regulating glutamate cysteine ligase [31]. These findings could explain, in part, the major vulnerability of MYCN-amplified NB cells following treatment with BSO, an irreversible inhibitor of GSH biosynthesis.

In this study, we provide evidence that PKCδ overexpression sensitizes MYCN-non amplified-NB cell lines to GSH depletion, inducing DNA oxidative damage and cell death. In SK-N-BE-2C, chosen as a model of MYCN-amplified cells, PKCδ overexpression is capable of potentiating the cytotoxic effects induced by BSO.

It has been established that ROS can act through several signaling molecules such as calcium, phospholipases, MAP kinases and PKC isozymes [32], [33]. PKCs are particularly important as they are part of the cell signaling machinery and contain regions, rich in cysteine residues, that are susceptible to redox modifications, both in the N-terminal regulatory domain and in the C-terminal catalytic domain [34], [35]. In particular PKCδ, a major isoenzyme ubiquitously expressed in most mammalian cells, is known for its apoptotic properties [36] and is involved in the cellular response to DNA damage and oxidative stress [37].

In the present study, we demonstrate that overexpression of active PKCδ, phosphorylated at Thr505, plays an essential role in the ROS signaling pathway leading to DNA damage and apoptosis of neuroblastoma cells. In fact, the blocking of ROS production by DPI prevented apoptosis of PKCδ-overexpressing cells depleted of GSH. Since DPI is a NADPH oxidase inhibitor [38] and several studies have demonstrated that PKC has a central role in the regulation of NADPH oxidase [39], [40], modulating the phosphorylation and activation of p67phox [41] and p47phox components [16], it is reasonable to assume that PKCδ acts upstream of ROS production, DNA oxidation and apoptosis. However, the possibility that free radicals are required for the activation of PKCδ cannot be completely excluded, even though neither PKC phosphorylation at Thr505 nor histone phosphorylation are significantly modified by BSO and/or DPI incubations. Only in SK-N-BE-2C wild-type cells, which have an increased basal level of free radicals, BSO exposure stimulates the kinase activity, as previously reported [30].

The initiating role of active PKCδ in the commitment to apoptosis of NB cells is suggested by the enhanced sensitivity of PKCδ-overexpressing cells to etoposide, and confirmed by the fact that rottlerin, which inhibits PKCδ by competing with ATP [42], effectively suppresses apoptosis and DNA damage in the same cells. Since rottlerin has been described as a factor having both PKCδ-dependent and -independent effects [43], [44] **,** we were prompted to apply targeted PKCδ mRNA silencing to clarify the direct role of PKCδ in the sensitization of neuroblastoma cells to the employed drugs. Our results show that PKCδ gene silencing, obtained by siRNA, completely blocked the induction of oxidative death and genotoxicity, observed in etoposide-treated SH-SY-5Y and in BSO-treated SK-N-BE-2C cells. In addition, we demonstrate that the overexpression of PKCδ-DN is incapable of potentiating the genotoxic effects induced by BSO and etoposide. Collectively, these molecular approaches (siRNA and PKCδ-DN) give independent evidence confirming that neuroblastoma cells need a functionally active PKCδ in order to become sensitive to these drugs. In fact, we have previously demonstrated [30], and confirmed here, that BSO induced apoptosis only in neuroblastoma cells in which it activates PKCδ.

In line with our data, other studies have shown that PKCδ deficient cells are impaired in their response to genotoxic agents, and that this kinase is required in order to trigger apoptosis after stress stimuli [45], [46].

Insight into how PKCδ may regulate apoptosis has been gained from studies that have investigated its subcellular localization in apoptotic cells. TPA, a well-known oxidant tumor promoter [47] induces translocation of PKCδ from the cytoplasm to mitochondria and this translocation results in the release of cytochrome c and the activation of caspase-3 [48]. Moreover, PKCδ translocates to the nucleus in response to ionizing radiation and chemotherapeutic agents [49].

Recent studies have shown that nuclear translocation of full-length PKCδ is required for the initiation of apoptosis induced by etoposide [50] and that apoptosis of SK-N-AS neuroblastoma cells, by caspase-dependent cleavage of PKCδ, is promoted by highly cytotoxic concentrations of etoposide [51].

In our study, treatment of NB cells with a low dose of etoposide causes oxidative DNA damage that is increased after PKCδ overexpression. Treatment with etoposide and BSO (1, 3 and 6 h) induces, in PKCδ-overexpressing cells, an early and transitory translocation of active full-length PKCδ to the nuclear compartment. This event, that preceded the appearance of apoptosis, is required to induce the late DNA damage. In fact, when cells were transfected with PKCδ-DN, the BSO or etoposide exposure did not cause the nuclear localization of PKCδ and there was no evidence of DNA damage. This demonstrates that it is only the catalytically competent kinase that is able to translocate to the nucleus and induce or amplify the genotoxic damage. Moreover, incubation with etoposide and BSO (1, 3 and 6 h) is not accompanied by the appearance of the catalytic fragment of PKCδ. Therefore, our results show that PKCδ-nuclear translocation is an essential key step to prime the sequence of molecular events which culminate in the late caspase 3 activation and in the irreversible commitment of cells to apoptosis. Accordingly, it has been demonstrated that nuclear accumulation of PKCδ is caspase-independent and occurs prior to activation of the components of the apoptotic pathway [52].

Numerous PKCδ targets and substrates are nuclear proteins that function in apoptotic cell death, such as the nuclear DNA-dependent protein kinase (DNA-PK) which plays an essential role in the repair of DNA double-strand breaks [53]. It has been demonstrated that activated PKCδ phosphorylates DNA-PK and induces its dissociation from DNA, inhibiting DNA repair and enhancing DNA fragmentation [54]. These findings are in agreement with the role of PKCδ overexpression in the DNA damage in consequence to BSO or etoposide treatment.

In this context, a thorough understanding of how PKCδ regulates apoptosis, in particular when cancer cells acquire resistance to chemotherapeutic drugs, could significantly improve the efficiency of cancer therapy.

Altogether, our data provides information for a potential PKCδ-based therapy that, in combination with chemotherapeutic agents, could act as a chemo-sensitizer, reducing drug dosages and relative periods of treatment and preventing the development of chemoresistance.

In conclusion, to tailor a specific therapy, we believe that analyses of ROS-mediated survival pathways, taking account of the tumor type and stage, might be helpful.

## References

[1] Fulda S, Debatin, KM (2003). Apoptosis pathways in neuroblastoma therapy.. Cancer Lett.

[2] de Bernardo S, Canals S, Casarejos MJ, Solano RM, Menendez J (2004). Role of extracellular signal-regulated protein kinase in neuronal cell death induced by glutathione depletion in neuron/glia mesencephalic cultures.. J Neurochem.

[3] Nishizuka Y (1992). Intracellular signaling by hydrolysis of phospholipids and activation of protein kinase C. Science.

[4] Cacace AM, Ueffing M, Philipp A, Han EK, Kolch W (1996). PKC epsilon functions as an oncogene by enhancing activation of the Raf kinase.. Oncogene.

[5] Heit I, Wieser RJ, Herget T, Faust D, Borchert-Stuhltrager M (2001). Involvement of protein kinase Cdelta in contact-dependent inhibition of growth in human and murine fibroblasts.. Oncogene.

[6] Hornia A, Lu Z, Sukezane T, Zhong M, Joseph T (1999). Antagonistic effects of protein kinase C alpha and delta on both transformation and phospholipase D activity mediated by the epidermal growth factor receptor.. Mol Cell Biol.

[7] Jiang XH, Tu SP, Cui JT, Lin MC, Xia HH (2004). Antisense targeting protein kinase C alpha and beta1 inhibits gastric carcinogenesis.. Cancer Res.

[8] Perletti GP, Marras E, Concari P, Piccinini F, Tashjian AH (1999). PKCdelta acts as a growth and tumor suppressor in rat colonic epithelial cells.. Oncogene.

[9] Kambhampati S, Li Y, Verma A, Sassano A, Majchrzak B (2003). Activation of protein kinase C delta by all-trans-retinoic acid.. J Biol Chem.

[10] Emoto Y, Kisaki H, Manome Y, Kharbanda S, Kufe D (1996). Activation of protein kinase Cdelta in human myeloid leukemia cells treated with 1-beta-D-arabinofuranosylcytosine.. Blood.

[11] Jackson DN, Foster DA (2004). The enigmatic protein kinase Cdelta: complex roles in cell proliferation and survival.. Faseb J.

[12] Marengo B, Raffaghello L, Pistoia V, Cottalasso D, Pronzato MA (2005). Reactive oxygen species: biological stimuli of neuroblastoma cell response.. Cancer Lett.

[13] Simon T, Langler A, Harnischmacher U, Fruhwald MC, Jorch N (2007). Topotecan, cyclophosphamide, and etoposide (TCE) in the treatment of high-risk neuroblastoma. Results of a phase-II trial.. J Cancer Res Clin Oncol.

[14] Hsieh HL, Wang HH, Wu CY, Jou MJ, Yen MH (2007). BK-induced COX-2expression via PKC-δ-dependent activation of p42/p44 MAPK and NF-kB in astrocytes.. Cell Signal.

[15] Domenicotti C, Marengo B, Verzola D, Garibotto G, Traverso N (2003). Role of PKC-delta activity in glutathione-depleted neuroblastoma cells.. Free Radic Biol Med.

[16] Nitti M, Furfaro AL, Traverso N, Odetti P, Storace D (2007). PKC delta and NADPH oxidase in AGE-induced neuronal death.. Neurosci Lett.

[17] Pessino A, Passalacqua M, Sparatore B, Patrone M, Melloni E (1995). Antisense oligodeoxynucleotide inhibition of delta protein kinase C expression accelerates induced differentiation of murine erythroleukaemia cells.. Biochem J.

[18] Ellerby LM, Bredesen DE (2000). Measurement of cellular oxidation, reactive oxygen species, and antioxidant enzymes during apoptosis.. Methods Enzymol.

[19] Cavallo D, Ursini CL, Carelli G, Iavicoli I, Ciervo A (2006). Occupational exposure in airport personnel: characterization and evaluation of genotoxic and oxidative effects.. Toxicology.

[20] Reyland ME, Anderson SM, Matassa AA, Barzen KA, Quissell DO (1999). Protein kinase C delta is essential for etoposide-induced apoptosis in salivary gland acinar cells.. J Biol Chem.

[21] Blass M, Kronfeld I, Kazimirsky G, Blumberg PM, Brodie C (2002). Tyrosine phosphorylation of protein kinase Cdelta is essential for its apoptotic effect in response to etoposide.. Mol Cell Biol.

[22] Karlsson J, Ora I, Porn-Ares I, Pahlman S (2004). Arsenic trioxide-induced death of neuroblastoma cells involves activation of Bax and does not require p53.. Clin Cancer Res.

[23] Gschwendt M, Muller HJ, Kielbassa K, Zang R, Kittstein W (1994). Rottlerin, a novel protein kinase inhibitor.. Biochem Biophys Res Commun.

[24] Li W, Yu JC, Michieli P, Beeler JF, Ellmore N (1994). Stimulation of the platelet-derived growth factor beta receptor signaling pathway activates protein kinase C-delta.. Mol Cell Biol.

[25] Friesen C, Kiess Y, Debatin KM (2004). A critical role of glutathione in determining apoptosis sensitivity and resistance in leukemia cells.. Cell Death Differ.

[26] Anderson CP, Tsai JM, Meek WE, Liu RM, Tang Y (1999). Depletion of glutathione by buthionine sulfoxine is cytotoxic for human neuroblastoma cell lines via apoptosis.. Exp Cell Res.

[27] Puhakka A, Ollikainen T, Soini Y, Kahlos K, Saily M (2002). Modulation of DNA single-strand breaks by intracellular glutathione in human lung cells exposed to asbestos fibers.. Mutat Res.

[28] Reliene R, Schiestl RH (2006). Glutathione depletion by buthionine sulfoximine induces DNA deletions in mice.. Carcinogenesis.

[29] Gokce G, Ozsarlak-Sozer G, Oktay G, Kirkali G, Jaruga P (2009). Glutathione depletion by buthionine sulfoximine induces oxidative damage to DNA in organs of rabbits in vivo.. Biochemistry.

[30] Marengo B, De Ciucis C, Verzola D, Pistoia V, Raffaghello L (2008). Mechanisms of BSO (L-buthionine-S,R-sulfoximine)-induced cytotoxic effects in neuroblastoma.. Free Radic Biol Med.

[31] de Tudela MV, Delgado-Esteban M, Cuende J, Bolanos JP, Almeida A (2010). Human neuroblastoma cells with MYCN amplification are selectively resistant to oxidative stress by transcriptionally up-regulating glutamate cysteine ligase.. J Neurochem.

[32] Herrlich P, Bohmer FD (2000). Redox regulation of signal transduction in mammalian cells.. Biochem Pharmacol.

[33] Gopalakrishna R, Jaken S (2000). Protein kinase C signaling and oxidative stress.. Free Radic Biol Med.

[34] Gopalakrishna R, Anderson WB (1987). Susceptibility of protein kinase C to oxidative inactivation: loss of both phosphotransferase activity and phorbol diester binding.. FEBS Lett.

[35] Gopalakrishna R, Anderson WB (1989). Ca2+- and phospholipid-independent activation of protein kinase C by selective oxidative modification of the regulatory domain.. Proc Natl Acad Sci U S A.

[36] Basu A, Woolard MD, Johnson CL (2001). Involvement of protein kinase C-delta in DNA damage-induced apoptosis.. Cell Death Differ.

[37] Brodie C, Blumberg PM (2003). Regulation of cell apoptosis by protein kinase c delta.. Apoptosis.

[38] Anantharam V, Kaul S, Song C, Kanthasamy A, Kanthasamy AG (2007). Pharmacological inhibition of neuronal NADPH oxidase protects against 1-methyl-4-phenylpyridinium (MPP+)-induced oxidative stress and apoptosis in mesencephalic dopaminergic neuronal cells.. Neurotoxicology.

[39] Bey EA, Xu B, Bhattacharjee A, Oldfield CM, Zhao X (2004). Protein kinase C delta is required for p47phox phosphorylation and translocation in activated human monocytes.. J Immunol.

[40] Nitti M, Furfaro AL, Cevasco C, Traverso N, Marinari UM (2010). PKC delta and NADPH oxidase in retinoic acid-induced neuroblastoma cell differentiation.. Cell Signal.

[41] Zhao X, Xu B, Bhattacharjee A, Oldfield CM, Wientjes FB (2005). Protein kinase Cdelta regulates p67phox phosphorylation in human monocytes.. J Leukoc Biol.

[42] Soltoff SP (2001). Rottlerin is a mitochondrial uncoupler that decreases cellular ATP levels and indirectly blocks protein kinase Cdelta tyrosine phosphorylation.. J Biol Chem.

[43] Davies SP, Reddy H, Caivano M, Cohen P (2000). Specificity and mechanism of action of some commonly used protein kinase inhibitors.. Biochem J.

[44] Soltoff SP (2007). Rottlerin: an inappropriate and ineffective inhibitor of PKCdelta.. Trends Pharmachol Sci.

[45] Leitges M, Mayr M, Braun U, Mayr U, Li C (2001). Exacerbated vein graft arteriosclerosis in protein kinase Cdelta-null mice.. J Clin Invest.

[46] Li PF, Maasch C, Haller H, Dietz R, von Harsdorf R (1999). Requirement for protein kinase C in reactive oxygen species-induced apoptosis of vascular smooth muscle cells.. Circulation.

[47] Wu WS (2006). The signaling mechanism of ROS in tumor progression.. Cancer Metastasis Rev.

[48] Lynch K, Fernandez G, Pappalardo A, Peluso JJ (2000). Basic fibroblast growth factor inhibits apoptosis of spontaneously immortalized granulosa cells by regulating intracellular free calcium levels through a protein kinase Cdelta-dependent pathway.. Endocrinology.

[49] DeVries TA, Neville MC, Reyland ME (2002). Nuclear import of PKCdelta is required for apoptosis: identification of a novel nuclear import sequence.. Embo J.

[50] DeVries-Seimon TA, Ohm AM, Humphries MJ, Reyland ME (2007). Induction of apoptosis is driven by nuclear retention of protein kinase C delta.. J Biol Chem.

[51] Day TW, Wu CH, Safa AR (2009). Etoposide induces protein kinase Cdelta- and caspase-3-dependent apoptosis in neuroblastoma cancer cells.. Mol Pharmacol.

[52] Reyland ME (2007). Protein kinase C delta and apoptosis.. Biochem Soc Trans.

[53] Bharti A, Kraeft SK, Gounder M, Pandey P, Jin S (1998). Inactivation of DNA-dependent protein kinase by protein kinase Cdelta: implications for apoptosis.. Mol Cell Biol.

[54] Yoshida K (2008). Nuclear trafficking of pro-apoptotic kinases in response to DNA damage.. Trends Mol Med.

